# Administration of SB239063 Ameliorates Ovariectomy-Induced Bone Loss *via* Suppressing Osteoclastogenesis in Mice

**DOI:** 10.3389/fphar.2019.00900

**Published:** 2019-08-15

**Authors:** Bao Huang, Jiasheng Wang, Xuyang Zhang, Ziang Xie, Hao Wu, Junhui Liu, Zhiwei Jie, Xiangde Zhao, An Qin, Shunwu Fan, Jian Chen, Fengdong Zhao

**Affiliations:** ^1^Department of Orthopaedic Surgery, Sir Run Run Shaw Hospital, Zhejiang University School of Medicine, Hangzhou, China; ^2^Key Laboratory of Musculoskeletal System Degeneration and Regeneration Translational Research of Zhejiang Province, Hangzhou, China; ^3^Department of Orthopaedics, Shanghai Key Laboratory of Orthopaedic Implant, Shanghai Ninth People’s Hospital, Shanghai Jiao Tong University School of Medicine, Shanghai, China

**Keywords:** SB239063, osteoclastogenesis, c-Fos, MEF2C, p38, bone loss

## Abstract

Activation of osteoclast formation and function is crucial for the development of osteolytic diseases such as osteoporosis. RANKL (receptor activator of nuclear factor-κB ligand) activates NF-κB (nuclear factor κB), MAPK (mitogen-activated protein kinase), and NFATc1 (nuclear factor of activated T-cells, cytoplasmic 1) signaling pathways to induce osteoclastogenesis. In this study, we demonstrated that SB239063, a p38-specific inhibitor, suppressed osteoclastogenesis and bone resorption *via* inhibiting phosphorylation of MEF2C (myocyte enhancer factor 2C) and subsequently leading to MEF2C degradation by ubiquitination. Knockdown of MEF2C impaired osteoclast formation due to decreased c-Fos expression. Furthermore, MEF2C can directly bind to the promoter region of c-Fos to initiate its transcription. Interestingly, overexpression of either MEF2C or c-Fos can partially rescue the inhibitory effect of SB239063 on osteoclastogenesis. In addition, *in vivo* data proved that SB239063 also played a preventive role in both LPS (lipopolysaccharide)- and OVX (ovariectomy)-induced bone loss in mice. In conclusion, our results show that SB239063 can be a potential therapy for osteolytic diseases, and a novel p38/MEF2C/c-Fos axis is essential for osteoclastogenesis.

## Introduction

Bone is a highly dynamic tissue that undergoes consistent remodeling, which relies on the balance between bone-resorbing osteoclasts and bone-forming osteoblasts. Intriguingly, bone remodeling is generally deteriorative in typical lytic bone diseases such as osteoporosis, rheumatoid osteoarthritis, and cancer bone metastases. The receptor activator of nuclear factor-κ B ligand (RANKL) and its receptor [receptor activator of nuclear factor κ B (RANK)] are vital regulators in the formation and function of multinucleated osteoclasts (Arai et al., 1999), which are derived from the monocyte/macrophage lineage (Ash et al., 1980; Scheven et al., 1986). RANKL binding to RANK can lead to the activation of many molecules such as TRAF6 (TNF receptor-associated factor 6) (Darnay et al., 1999), following the activation of downstream signaling mechanisms including NF-κB (nuclear factor κB) and MAPK (mitogen-activated protein kinase) pathways (Pearson et al., 2001; Xie et al., 2018). Activation of p38/MAPK positively regulates the osteoclastogenesis. The multinucleated osteoclasts are gradually induced after upregulation and activation of downstream molecules, such as NFATc1 (nuclear factor of activated T-cells, cytoplasmic 1) (Takayanagi et al., 2002a) and c-Fos (Monje et al., 2005). Therefore, key molecules of the RANKL-induced signaling pathways could be therapeutic targets when treating osteoclastic diseases.

The activating protein-1 (AP-1) is a dimer consisted of c-Jun and c-Fos whose activation and expression are tightly regulated by the MAPK family (Treisman, 1996; Karin et al., 1997). Although the suppression of MAPK family could inhibit the c-Fos expression (Deepak et al., 2015; Chawalitpong et al., 2016), its exact mechanism remains unclear. Reversible phosphorylation of transcription factors may lead to changes in nuclear localization, negative or positive modulation of transactivation, rate of binding to target DNA sequences, and their stability (Hill and Treisman, 1995). However, it is still unclear if p38 participates in these interactions during the osteoclastogenesis.

The p38 has been shown to directly phosphorylate myocyte-specific enhancer factor 2C (MEF2C) that leads to its activation. MEF2C, also known as MADS box transcription enhancer factor 2 polypeptide C (Han et al., 1997; Zhang et al., 2014; Brown et al., 2018), plays a fundamental role in cell division, death, and differentiation by mediating DNA dimerization, binding, and interacting with transcriptional cofactors (Feng et al., 2009; Cante-Barrett et al., 2014). Interestingly, MEF2C deletion in osteocytes leads to increased bone mass (Kramer et al., 2012). MEF2C is vital for MMP13 expression by increased binding to c-Fos at the AP-1 site in MMP13 promotor region (Nakatani and Partridge, 2017). Nevertheless, the exact mechanisms through which MEF2C regulates these transcription factors during osteoclastogenesis remain unclear. Furthermore, SB239063 (a p38 specific inhibitor) is a novel, highly selective and specific inhibitor compared with the SB203580 (a p38 inhibitor). SB239063 alleviates acute lung injury (Xiong et al., 2016) and is a therapeutic target for hepatic encephalopathy (Agusti et al., 2011). However, its function during bone homeostasis is still unknown. The basic goal of the present study is to apply SB239063 to the treatment for bone loss while exploring the effects of MEF2C regulated by p38/MAPK and illuminating the potential molecular mechanism on bone homeostasis.

## Materials and Methods

### Reagents

SB239063, a p38 specific inhibitor, was purchased from Selleck. Alpha-MEM, FBS (fetal bovine serum), and penicillin/streptomycin were obtained from Gibco. Cell counting kit-8 (CCK-8) was purchased from Multi Sciences (Hangzhou, China). Recombinant mouse MCSF and RANKL were purchased from R&D. DMSO concentration in medium was less than 1‰ of the total volume. Specific antibodies against p38, phospho-p38(Thr180/Tyr182), ERK, phospho-ERK (Thr202/Tyr204), JNK, phospho-JNK (Thr183/Tyr185), p65, phosphor-p65 (Ser536), IκB-α, NFATc1, c-Fos, nucleolin, and ubiquitin were purchased from CST. Specific antibodies against MEF2C, phospho-MEF2C (Ser396), α-tubulin, β-actin, and GAPDH were purchased from Abcam. Antibody against MEF2C for ChIP assay was also obtained from Proteintech. The osteoclasts were stained using TRAP (tartrate-resistant acid phosphatase) staining kits (Sigma-Aldrich).

### BMM Preparation and Differentiation

The BMMs (bone marrow monocytes) were collected from femoral and tibial bone marrow of C57BL/6 mice (male, 6 weeks old) as described previously (Xie et al., 2018). Briefly, bone marrow cells were isolated from the femurs and tibias and cultured with 10% FBS, alpha-MEM, 1% penicillin/streptomycin, and 25 ng/ml MCSF under 5% CO_2_, 37°C. BMMs were seeded in 96-well plates (0.6 × 10^4^ cells/well) with or without 25 ng/ml MCSF, 50 ng/ml RANKL, and different concentrations of SB239063 (12.5, 25, 50, 100, 200, and 400 nM). Multinucleated osteoclasts that were induced for 5–7 days were immediately fixed in 4% paraformaldehyde for 30 min at room temperature (RT) and stained in TRAP solution for 1 h at 37°C. TRAP-positive cells with more than three nuclei were considered osteoclasts. For F-actin ring staining, cells were cultured in the 96-well plates with or without SB239063 for 7 days. Osteoclasts were then fixed in 4% paraformaldehyde, permeabilized in 0.15% Triton X-100, washed in PBS, and immunostained with phalloidin (Servicebio).

### Cytotoxicity Assay

The cytotoxicity of SB239063 on BMMs was detected using the CCK-8 assay. The BMMs were seeded in 96-well plates (1 × 10^4^ cells/well) in quadruplicate with 25 ng/ml MCSF overnight. Cells were then treated with indicated concentration of SB239063 (0, 0.1, 0.2, 0.4, 0.8, 1.6, 3.2, and 6.4 μM) for 48 or 96 h. Afterward, a 10-μl CCK-8 buffer was added to each well. All plates were incubated at 37°C for an additional 1 h. Absorbance of 450-nm wavelength was detected using the Multiscan Go (Thermo Scientific).

### Bone Resorption Assay

The BMM-derived pre-osteoclasts that were treated with MCSF and RANKL to form osteoclasts over 3 days were seeded on bovine bone slices (Shenggong, Shanghai, China) in a 96-well plate (1 × 10^4^ cells/well), in triplicate. The cells were then stimulated with or without SB239063 (0, 25, 50, 100, 200, or 400 nM) for over 3 days. Cells on the surface of bovine bone slices were then slightly brushed away. The pits on the slices were scanned by a SEM (TM-1000, Hitachi, Japan). Pits area was quantified by Image J.

### Reverse Transcription and Quantitative PCR

The cells were seeded (2 × 10^5^ cells/well) in 12-well plates and cultured with 25 ng/ml MCSF overnight. BMMs were then stimulated with 200-nM SB239063 and 50 ng/ml RANKL for 0–5 days. All the RNAs of cells were extracted using Ultrapure RNA Kit (CW0581, CWBIO, China) according to the manufacturer’s protocols. Quantity of RNAs was measured by Nanodrop 2000. Reverse transcription was conducted using HiFiScript cDNA Synthesis kit (CW2569, CWBIO, China). Then, RT-qPCR quantified transcription levels using the UltraSYBR Mixture (CW0957, CWBIO, China) according to the manufacturer’s protocols. Experimental reactions were conducted at 95°C for 10 min (preincubation), 95°C for 15 s, 60°C for 60 s, and 72°C for 20 s for 40 cycles (amplification) and 95°C for 15 s, 60°C for 60 s (melting curves), and 4°C for 5 min (cooling). Primer sequences of β-actin, GAPDH, c-Fos, NFATc1, MEF2C, TRAP, and CTSK were summarized in **Supplementary Table 1**.

### Western Blot Analysis

BMMs or RAW264.7 cells were seeded (4 × 10^5^ cells/well) in 6-well plates. Cells were pretreated with or without 200-nM SB2390663 for 3 h. Cells were then treated with 50 ng/ml RANKL for indicated time, with or without 200-nM SB239063 for 30 min or 48 h. All the proteins were obtained from cultured cells by using RIPA (Solar bio, Beijing, China) supplemented with 100-mM phenylmethanesulfonyl fluoride (Beyotime, Zhengzhou, China), 100×Phosphatase Inhibitor Cocktail (CWBIO, China), and Protease Inhibitor Cocktail (Millipore, USA). After the 15-min centrifugation at 12,000 rpm, supernatants were extracted. Proteins were resolved on 10% SDS-PAGE and transferred by electroblotting to PVDF membranes (Millipore, USA). Membranes were then blocked in 5% (w/v) nonfat dry milk in TBS with Tween 20 (TBST) at RT for 45 min, followed by incubation with indicated antibodies (1:1,000 dilution) at 4°C overnight. Washed five times with TBST, bands were then incubated with the HRP-conjugated goat anti-mouse/rabbit IgG (1:5,000 dilution; Abcam). Bands were detected by Image Lab software (Bio-Rad, Hercules, CA). The images were quantified by Image J.

### siRNAs Transfection and DNA Transduction

Primary BMMs were transfected with DNA as described previously (Xie et al., 2018). Given the same dose of lipofectamine 3000 reagent (0.45 μl for 96 well; 5 μl for 6 well), BMMs were separately incubated with siRNAs (Ribobio, Shanghai, China) specifically targeting MEF2C at the beginning (called 1st) or 48 h (called 3rd) after RANKL treatment. We always replaced the cultured medium after 24-h siRNA transfecting. As for DNA transduction, we first infected BMMs with the viral supernatants of pLenti-EF1a-MEF2C or pLenti-EF1a-c-Fos (Vigenebio, Shandong, China) for 6 h and after 48 h, transfected the siRNA, then treated with RANKL. BMMs were lysed to detect the MEF2C and c-Fos expression after 48-h transfecting or infecting. The differentiation of osteoclasts was then conducted with 25 ng/ml MCSF and 100 ng/ml RANKL. After 7 days, osteoclast formation was measured by TRAP staining. The siRNAs and DNA were summarized in **Supplementary Table 2**.

### Luciferase Reporter Assay

Briefly, HEK293 cells were transfected with a vector or p-c-Fos-luc (Vigene, Shandong, China) along with Renilla (Promega). Luciferase activity was measured using the dual luciferase reporter assay kit (Beyotime, Shanghai, China). Relative luciferase activity was normalized to Renilla.

### Chromatin Immunoprecipitation (ChIP)

ChIP assay was conducted using the ChIP assay kit (9002, CST), following the manufacturer’s instructions as described previously (Nakatani and Partridge, 2017). BMMs were stimulated with or without 50 ng/ml RANKL for 18 h and then incubated in 37% formaldehyde for 10 min. Washed three times in cold PBS with 1-mM PMSF and protease inhibitor cocktail, cells were then suspended in ChIP buffer with protease inhibitors and 1-mM PMSF for 10 min. Samples were then sonicated with 10 30-s pulses and 30-s intervals on ice. After centrifugation at 9,400×g for 10 min, supernatants were obtained and diluted with ChIP dilution buffer with 1-mM PMSF and protease inhibitors. Aliquots (1:100) of total chromatin DNA were preserved as input before immunoprecipitation. The supernatants precleared with protein A/G-agarose beads were collected for overnight incubation with MEF2C antibody at 4°C. Chromatin was eluted from the antibody/protein G agarose beads by vortexing at 1,200 rpm for 30 min at 65°C. Supernatants were obtained and incubated for 2 h at 65°C after the addition of 6-µl 5-M NaCl and 2-µl Proteinase K to reverse crosslinks. DNAs were purified by DNA spin column. DNAs was then amplified using PCR.

### Co-Immunoprecipitation (IP) Assay

Cell lysates were immunoprecipitated with 2-μg anti-MEF2C antibody at 4°C overnight, then incubated with protein A/G-agarose (50% v/v) for 3 h at 4°C. The protein A/G agarose–antigen–antibody complexes were collected after centrifugation at 16,000×g at 4°C for 1 min. Bound proteins were washed five times and resolved by 8% SDS-PAGE, then incubated with anti-MEF2C and anti-ubiquitin.

### Calvarial Osteolysis Model

This study was approved by the Ethics Committee of Sir Run Run Shaw Hospital (Zhejiang University affiliated, Hangzhou, Zhejiang). Calvarial osteolysis model was used to detect the effect of SB239063 on osteolysis *in vivo*. Briefly, 18 healthy C57BL/6 mice (male, 12 weeks old) were randomly distributed to three groups: the sham group (PBS injection), the vehicle group (LPS injection with PBS), and the SB239063 group (LPS injection and 4 mg/kg SB239063). Across the calvarial sagittal midline suture, the mice were subcutaneously injected with 25 mg/kg LPS (L2880, Sigma-Aldrich) once over a 15-day period under anesthesia. The mice were intraperitoneally injected with 4 mg/kg SB239063 every 3 days in 15 days. Craniums (parietal, occipital, and frontal bone) were then obtained and immediately fixed in 4% (w/v) paraformaldehyde for μ-CT and bone histomorphometry analyses.

### Ovariectomy (OVX)-Induced Osteoporosis Model

OVX-induced osteoporosis model was next established to assess the role of SB239063 in osteoporosis as previously described (Xie et al., 2018). Briefly, 18 healthy C57BL/6 mice (female, 12 weeks old) were randomly distributed to three groups: the sham group (sham operation and PBS injection), the vehicle group (OVX and PBS injection), and the SB239063 group (OVX and 4 mg/kg SB239063 injection). Mice in the SB239063 group were injected intraperitoneally with 4 mg/kg SB239063 twice a week in 8 weeks. Meanwhile, equivalent volume of PBS was injected in the sham group and the vehicle group. All the mice were then sacrificed. Mice uterus and body weights in the groups are shown in **Figures S2A, S2B**. Femurs and tibiae were immediately fixed in 4% (w/v) paraformaldehyde for μ-CT and bone histomorphometry analyses.

### µ-CT Analysis

Distal femurs and craniums were scanned by the μ-CT scanner (Skyscan 1072, Aartselaar, Belgium). According to the protocol, we used the X-ray energy of 80 μA/70 kV and the isometric resolution of 9 μm. The BV/TV (trabecular bone volume per total volume), mean Tb.Th (trabecular thickness), mean Tb.Sp (trabecular separation), and mean Tb.N (trabecular number) were analyzed by the reconstruction program, as described previously (Xie et al., 2018).

### Histological Analysis

Fixed femurs and tibiae were decalcified in 12% EDTA2Na at RT for approximately 3 weeks and embedded in paraffin, as previously described (Xie et al., 2018). Their slices were used for TRAP staining, H&E (hematoxylin and eosin) staining, and immunohistochemical staining. The number of TRAP-positive osteoclasts and OcS/BS (osteoclast surface area relative to bone surface area) were also assessed by a microscope.

### Statistical Analysis

The data were presented as mean ± SEM. Data analyses were conducted using SPSS 19.0 (SPSS, Chicago). Statistical differences were analyzed by one-way ANOVA or Student’s t-test, followed by Tukey’s *post hoc* analyses. P-value ≤ 0.05 was considered statistically significant.

## Results

### SB239063 Inhibits Osteoclastogenesis and Impairs Osteoclastic Bone Resorption *In Vitro*

**Figure 1:**
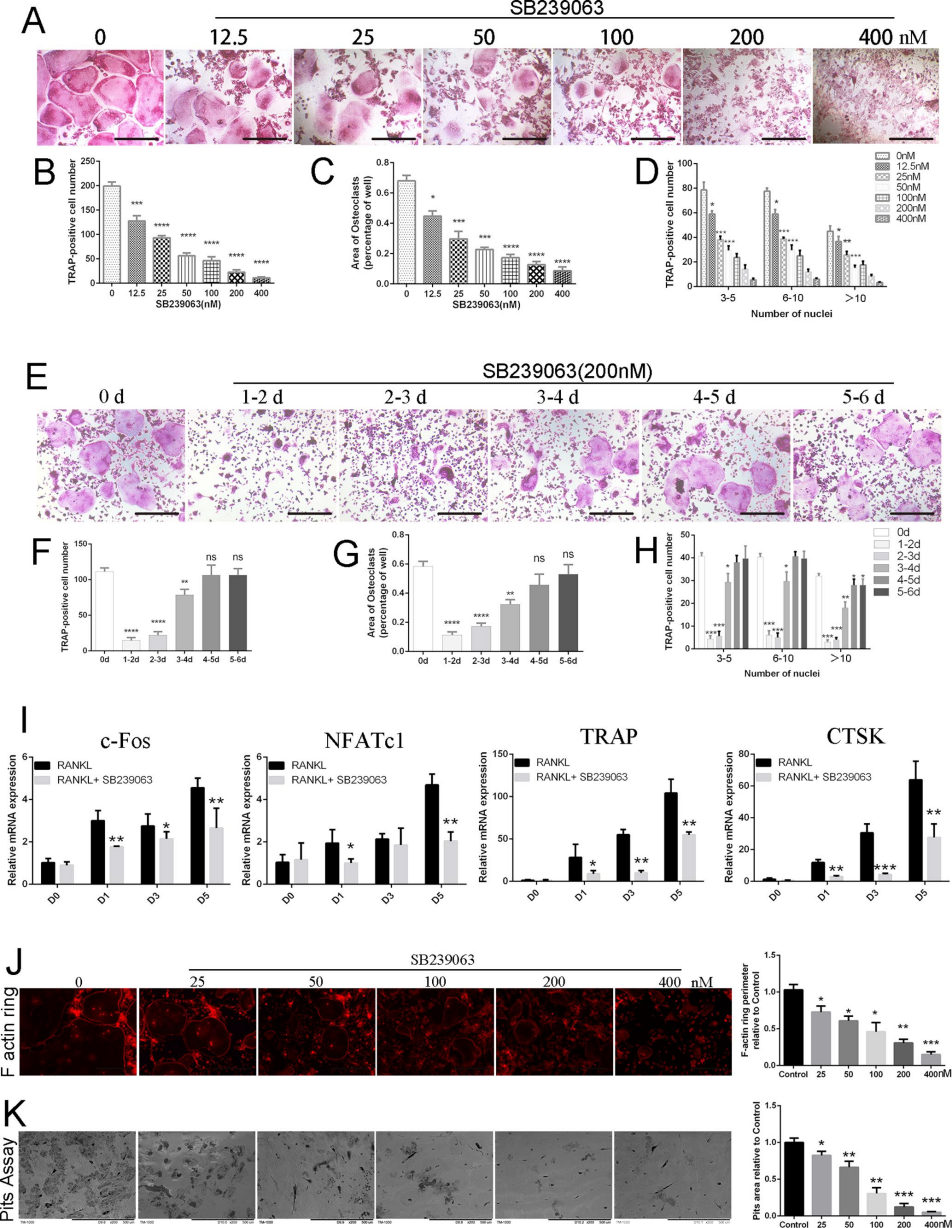
SB239063 inhibits osteoclastogenesis and impairs osteoclastic bone resorption *in vitro*. **(A)**TRAP-positive BMMs treated with different concentrations of SB239063 followed by the stimulation with M-CSF and RANKL. **(B–D)** Quantification of TRAP-positive multinuclear cells, **(B)** osteoclast number, **(C)** area, and **(D)** size of osteoclasts. **(E)** TRAP-positive BMMs following the treatment with 200-nM SB239063 for the indicated days during osteoclastogenesis. **(F–H)** Quantification of TRAP-positive multinuclear cells, **(F)** osteoclast number, **(G)** area, and **(H)** size of osteoclasts. **(I)** Gene expression of c-Fos, NFATc1, TRAP, and CTSK in BMMs treated with 200-nM SB239063 for the indicated time. **(J)** F-actin ring images were presented following the indicated concentrations of SB2339063 for 6 days, and (right) quantification of F actin ring perimeter (100%) relative to untreated group. **(K)** Bone resorption pit images were obtained by scanning electron microscope following the SB239063 treatment, and (right) quantification of pits area (100%) relative to untreated group. All experiments were performed at least three times. Scale bar, 100 µm. *P < 0.05, **P < 0.01, ***P < 0.005.

To investigate the potential cytotoxicity of SB239063 on BMMs, cell viability assay was conducted. As shown in **Figures S1B, S1C**, concentrations up to 0.8-μM SB239063 did not affect cell viability of BMMs, although concentrations 1.6 μM and above reduced viability. Surprisingly, SB239063 at the low concentration does not affect cell viability of osteoblasts (**Figures S2A, S2B**). However, osteoclasts formation following SB239063 treatment was significantly inhibited in a dose-dependent manner (11.0 ± 1.155 TRAP-positive multinucleated cells/well in 400-nM SB239063 group, while the number of TRAP-positive multinucleated osteoclasts was 199.0 ± 5.933 cells per well in control group) (**Figures 1A–D**). Furthermore, the number of osteoclasts was sharply reduced, while SB239063 treatment was applied in an early stage (days 1–3), but osteoclast formation was hardly affected with SB239063 treatment in the late stage (days 4–6) (**Figures 1E–H**). Although osteoclasts formation was not significantly suppressed, the area of multinucleated osteoclasts was slightly decreased with SB239063 treatment in the late stage (**Figure 1G**). Yet, osteoclasts with more than 10-cell nucleus were significantly reduced in the late treatment group (**Figure 1H**). The gene expression of CTSK, TRAP, c-Fos, and NFATc1 was suppressed during the osteoclast formation (**Figure 1I**). The inhibition of osteoclast-associated genes further supported the inhibitory role of SB239063 in osteoclasts formation *in vitro*. In addition, to determine the role of SB239063 in osteoclast function, BMMs were seeded on bovine bone slices and stimulated with indicated dose of SB239063. As shown in **Figures 1J**, **K**, SB239063 substantially reduced the perimeter of F-actin ring and bone resorption area in the dose-dependent manner. The area of bone resorption in 25-nM SB239063 treatment group was approximately 35%, compared with that in the control group. As expected, 400-nM SB239063 almost abolished bone resorption (**Figure 1K**). Together, these results suggest that SB239063 suppresses osteoclastogenesis *in vitro*.

### SB239063 Suppresses Osteoclastogenesis *via* Mainly Inhibiting the Nuclear Translocation of MEF2C *In Vitro*

**Figure 2:**
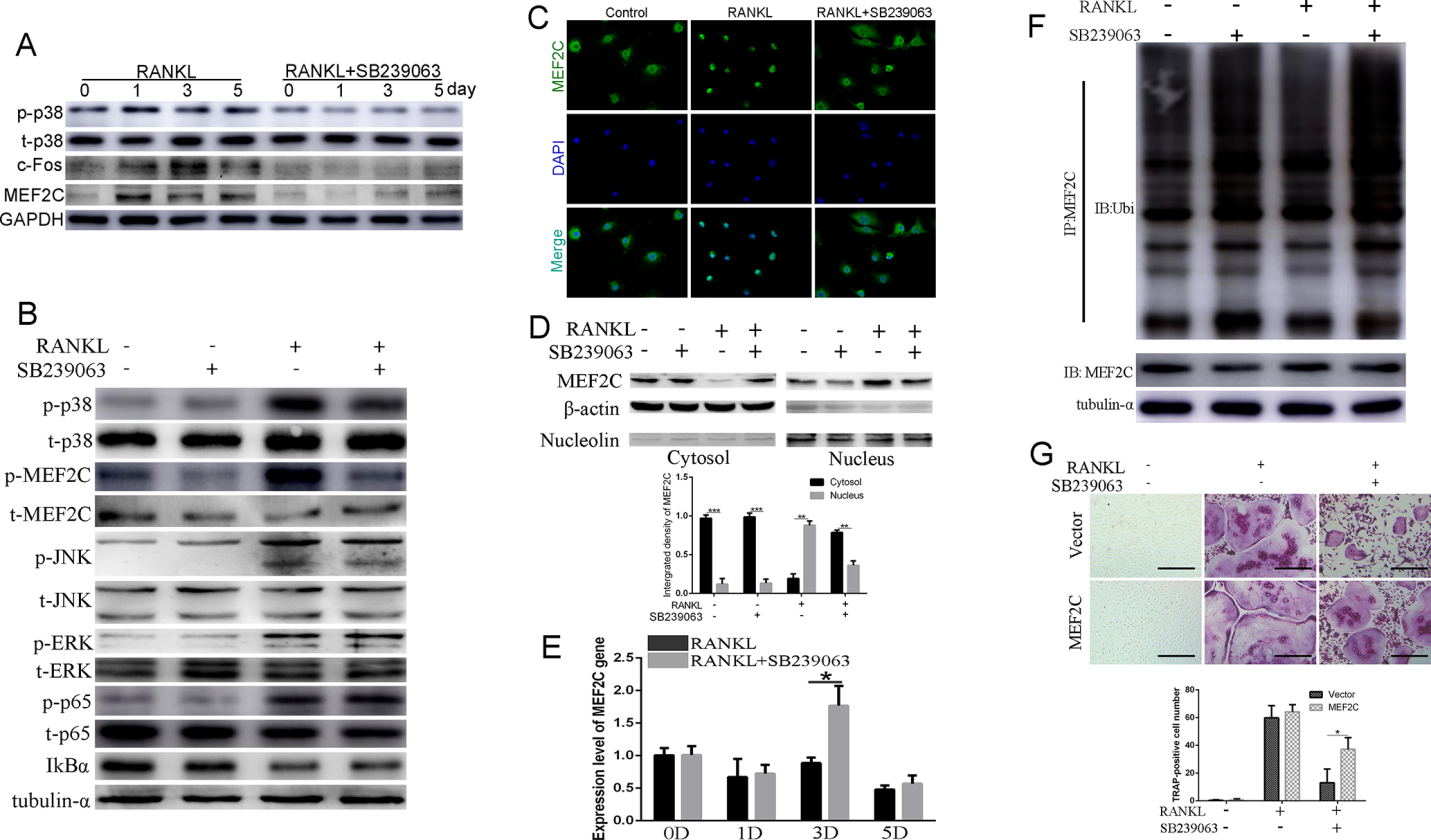
SB239063 suppresses osteoclastogenesis *via* mainly inhibiting the nuclear translocation of MEF2C *in vitro*. **(A)** Protein level of p-p38, MEF2C, and c-Fos in BMMs treated with or without SB239063 (200 nM) for 0, 1, 3, and 5 days. Untreated cells were used as control. **(B)** BMMs were pretreated with or without 200-nM SB239063 for 2 h before the stimulation with RANKL for 30 min. The expression of phosphorylated ERK, p38, JNK, p65, MEF2C, and total ERK, p38, JNK, p65, IκB-α, MEF2C, and tubulin-α was evaluated. **(C**–**D)** BMMs were pretreated with 200-nM SB239063 for 2 h before the stimulation with RANKL for 30 min. Nuclear translocation of MEF2C was visualized using immunofluorescence **(C)** and Western blotting assay **(D)**. **(E)** The mRNA level of MEF2C with or without SB239063 in the presence or absence of RANKL for the indicated periods in BMMs. **(F)** BMMs were incubated with RANKL and SB239063 or untreated for 18 h, and the immunoprecipitation with MEF2C antibody was performed. The obtained immunoprecipitates were analyzed using the indicated antibodies. **(G)** The images of TRAP-positive BMMs following the viral transduction of pLenti-EF1a-MEF2C or the empty vector and the treatment with SB239063. All experiments were performed at least three times. Scale bar, 100 µm. *P < 0.05, **P < 0.01, ***P < 0.005.

In view of the reports that MEF2C has been revealed to be a phosphorylation-dependent transcription factor mainly activated by p38/MAPK (Han et al., 1997; Zhang et al., 2014; Brown et al., 2018), we investigated whether SB239063 can suppress the nuclear translocation of MEF2C and subsequently influence c-Fos expression. Here, protein level of MEF2C was increased during osteoclastogenesis, which was consistent with the c-Fos (**Figure 2A**). SB239063 (200 nM) highly inhibited phosphorylation of MEF2C mainly by suppressing p38/MAPK (**Figures 2A, B**). Furthermore, immunofluorescence and Western blotting assay showed that SB239063 inhibited nuclear translocation of MEF2C (**Figures 2C, D**). Interestingly, mRNA level of MEF2C was not obviously decreased, while it could be increased at day 3 (**Figure 2E**). We assumed that MEF2C was modified at the protein level. As shown in **Figure 2F**, SB239063 treatment accelerated the ubiquitination of MEF2C that was then degraded. Furthermore, the constitutive activation of MEF2C (**Figure S1D**) partially rescued osteoclast formation (**Figure 2G**). Collectively, SB239063 inhibits the phosphorylation of MEF2C that plays an essential role in osteoclastogenesis.

### MEF2C Positively Regulates the Transcription of c-Fos During Osteoclastogenesis *In Vitro*

**Figure 3:**
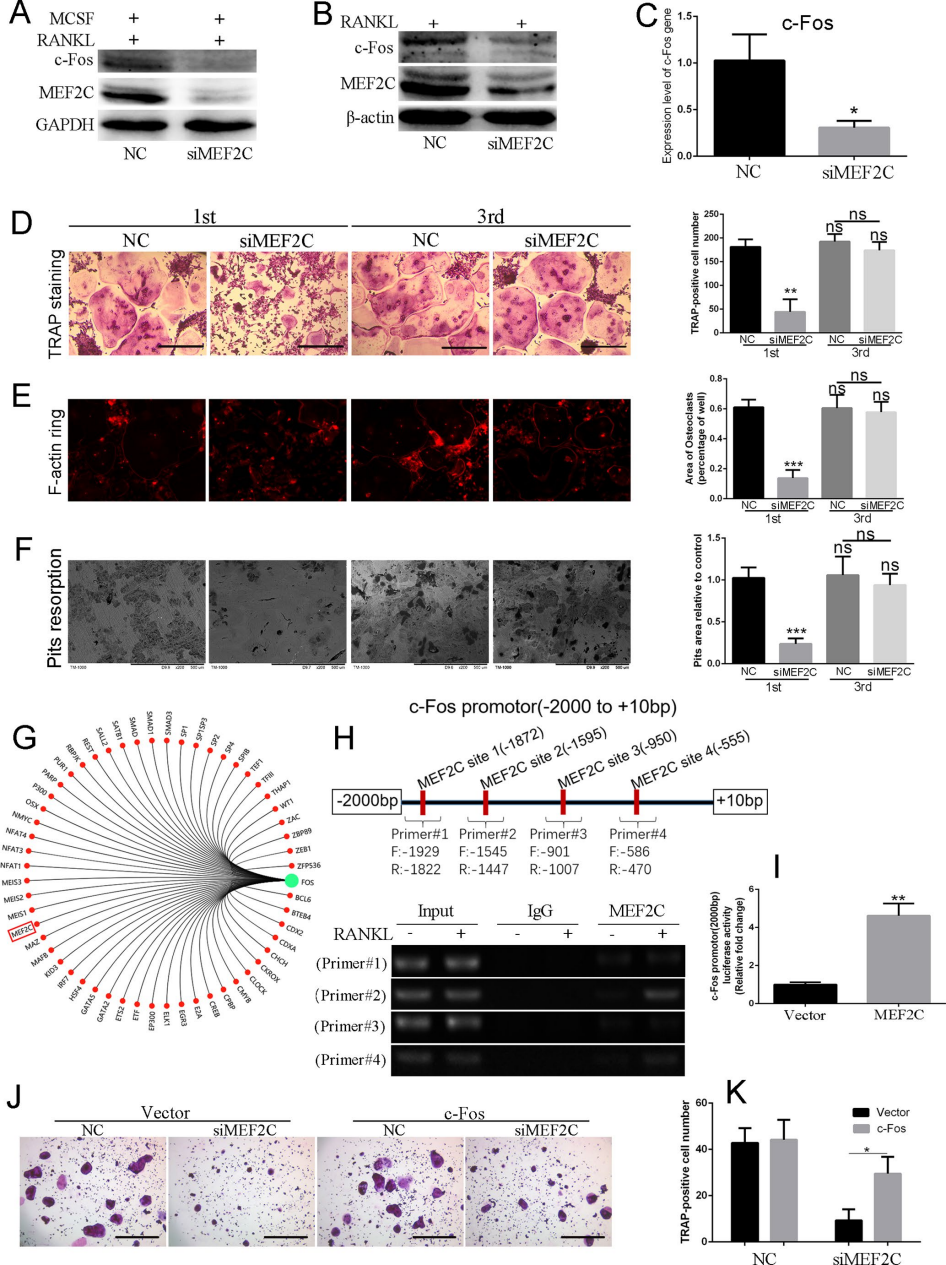
MEF2C positively regulates the transcription of c-Fos during osteoclastogenesis *in vitro*. **(A)** MEF2C and c-Fos expression after the knock down of MEF2C (siRNAs of MEF2C) for 48 h in BMMs and **(B)** RAW264.7 cells. **(C)** Gene expression level of c-Fos in BMMs treated with NC or siMEF2C. The typical images of TRAP staining **(D)**, F-actin ring staining **(E)**, and bone resorption **(F)** followed by incubation with or without the siMEF2C from the first day (1st) or third day (3rd), following 25 ng/ml MCSF and 100 ng/ml RANKL. **(G)** The transcriptional regulatory network of c-Fos by Gene-Cloud of Biotechnology Information. **(H)** Putative MEF2C binding sites within the 2,000-bp region of c-Fos promoter, and ChIP-PCR assay. **(I)** MEF2C increased transcriptional activity of c-Fos promotor compared with vector in HEK293 cells. **(J)** The constitutive activation of c-Fos partially rescued osteoclast formation. **(K)** Quantification of TRAP-positive multinuclear cells at **(J**). All experiments were performed at least three times. Scale bar, 100 µm. *P < 0.05, **P < 0.01, ***P < 0.005.

To identify the role of MEF2C in RANKL-induced multinucleated osteoclast, BMMs were stimulated with 25 ng/ml MCSF and 50 ng/ml RANKL with or without siMEF2C. Interestingly, knockdown of MEF2C suppressed RANKL-induced c-Fos expression in the BMMs (**Figure 3A**) and RAW264.7 cells at 48 h (**Figure 3B**). Also, mRNA level of c-Fos was significantly decreased after knockdown of MEF2C (**Figure 3C**). As shown in **Figures 3D, E**, the number, area, and the size of F-actin ring of TRAP-positive multinucleated osteoclasts were significantly decreased, while knockdown of MEF2C was applied in the early stage, while siMEF2C treatment resulted in considerable reduction of bone resorption area in the early treatment stage (**Figure 3F**). Only 23.72 ± 3.828% of bone resorption area was observed in the siMEF2C samples in the early treatment stage (**Figure 3F**). We then predicted the transcriptional regulatory network of c-Fos (**Figure 3G**) by Gene-Cloud of Biotechnology Information (GCBI: https://www.gcbi.com.cn) and further analyzed the putative MEF2C binding sites (**Figure 3H**) within the 2,000-bp c-Fos promoter region through the transcription element search software (JASPAR: http://jaspar.genereg.net). Furthermore, we investigated whether MEF2C interacted with the promoter region of c-Fos using ChIP assay. As shown in **Figure 3H**, MEF2C could increasingly bind to the sites of its promoter (−1,545 to −1,447 bp and −586 to −470bp) in BMMs after RANKL treatment, while binding to the sites (−1,939 to −1,822 bp and −1,007 to −901 bp) was not obviously elevated. Overexpression of MEF2C increased transcriptional activity of c-Fos 2,000-bp promoter by 4.617-fold compared with vector in HEK293 cells (**Figure 3I**). In addition, the constitutive activation of c-Fos (**Figure S1E**) partially rescued osteoclast formation, while the siMEF2C suppressed the osteoclastogenesis (**Figures 3J, K**). Finally, the constitutive activation of c-Fos could also partially counteract the inhibitory effect of SB239063 on osteoclast formation (**Figures S1F, S1G**). Taken together, MEF2C can positively control the c-Fos transcription during osteoclastogenesis.

### Administration of SB239063 Prevents LPS-Induced Osteolysis *In Vivo*

**Figure 4:**
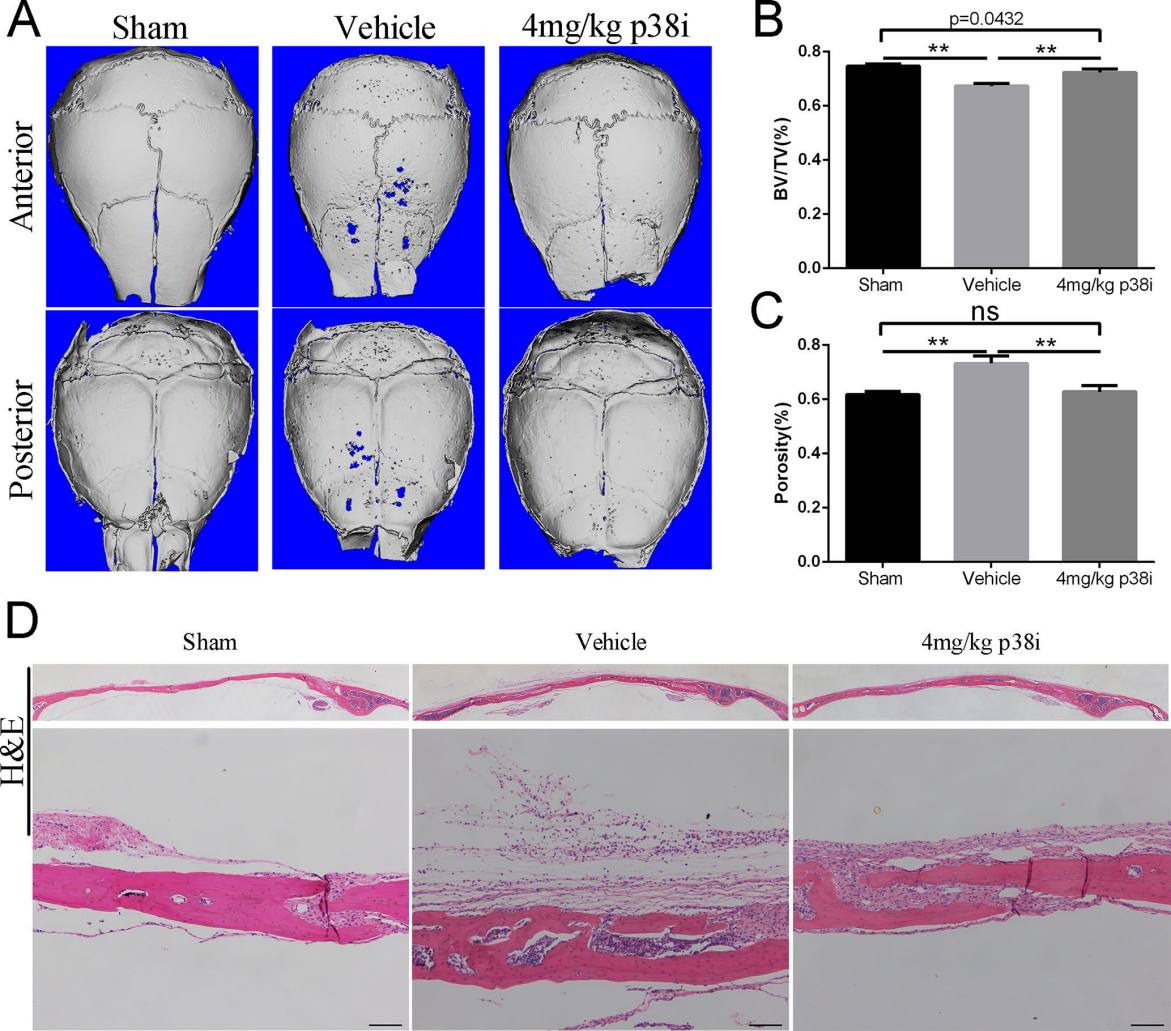
Administration of SB239063 prevents LPS-induced osteolysis *in vivo*. **(A)** Representative micro-CT and 3D reconstructed images were obtained for each group. **(B)** The BV/TV and **(C)** porosity of each sample were measured. The results were normalized to the sham group. **(D)** Effect of SB239063 on mouse LPS-induced calvarial osteolysis was accessed with H&E staining. Scale bars, 100 µm. *P < 0.05, **P < 0.01, ***P < 0.005.

When investigating the preventive effect of SB239063 on osteolytic diseases, the mice were subcutaneously injected with 25 mg/kg LPS (L2880, Sigma-Aldrich) once across the calvarial sagittal midline suture over a 15-day period under anesthesia. As expected, bone volume and porosity were significantly decreased by LPS treatment (**Figures 4A–C**). Treatment with SB239063 considerably increased bone volume and reduced porosity in the LPS treatment group (**Figures 4A–C**). Also, H&E staining revealed that osteolysis had clearly occurred in sections obtained from the vehicle group, whereas the SB239063 treatment groups exhibited significantly reduced osteolysis (**Figure 4D**). Together, we find out that administration of SB239063 prevents LPS-induced osteolysis *in vivo*.

### Administration of SB239063 Ameliorates OVX-Induced Bone Loss *In Vivo*

**Figure 5:**
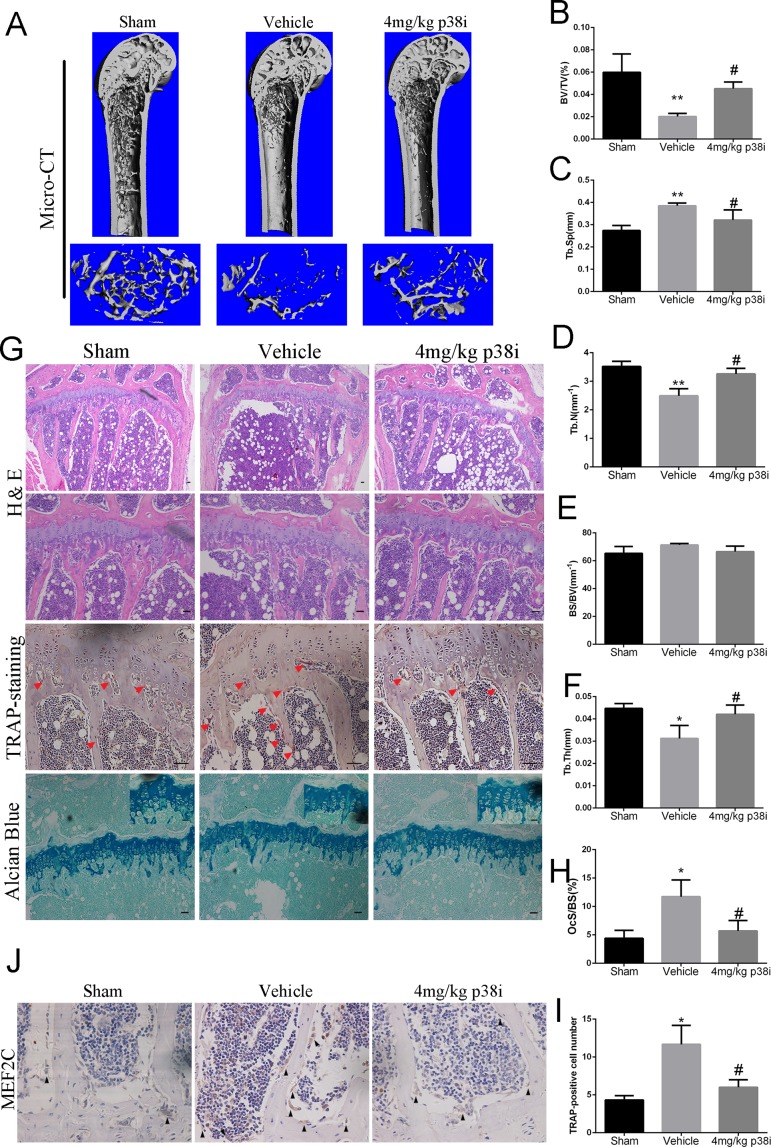
Administration of SB239063 ameliorates OVX-induced bone loss *in vivo*. **(A)** The right distal femurs of OVX mice divided into three groups and treated for 8 weeks were analyzed by micro-CT and three-dimensional reconstructed images are presented. **(B**–**F)** Bone volume/tissue volume (BV/TV), bone surface/bone volume (BS/BV), trabecular separation (Tb.Sp.), trabecular thickness (Tb.T.), and trabecular number (Tb.N.). obtained by analyzing the data from the three groups are presented. **(G)** H&E, TRAP staining (red arrows, TRAP-positive cells), and Alcian blue staining (growth plates) of the right proximal tibias. **(H)** The percentage of osteoclast surface per bone surface (OcS/BS%) and **(I)** the number of TRAP-positive cells per field of tissue in sections stained by TRAP in 200× magnification was analyzed. **(J)** MEF2C expression (black arrow) at the lumbar vertebral bodies (L3–L5) of three groups obtained by immunohistochemistry staining. Scale bars, 100 µm. #P < 0.05, *P < 0.05, **P < 0.01, ***P < 0.005 (#: sham versus vehicle group; *: vehicle versus treatment group).

**Figure 6:**
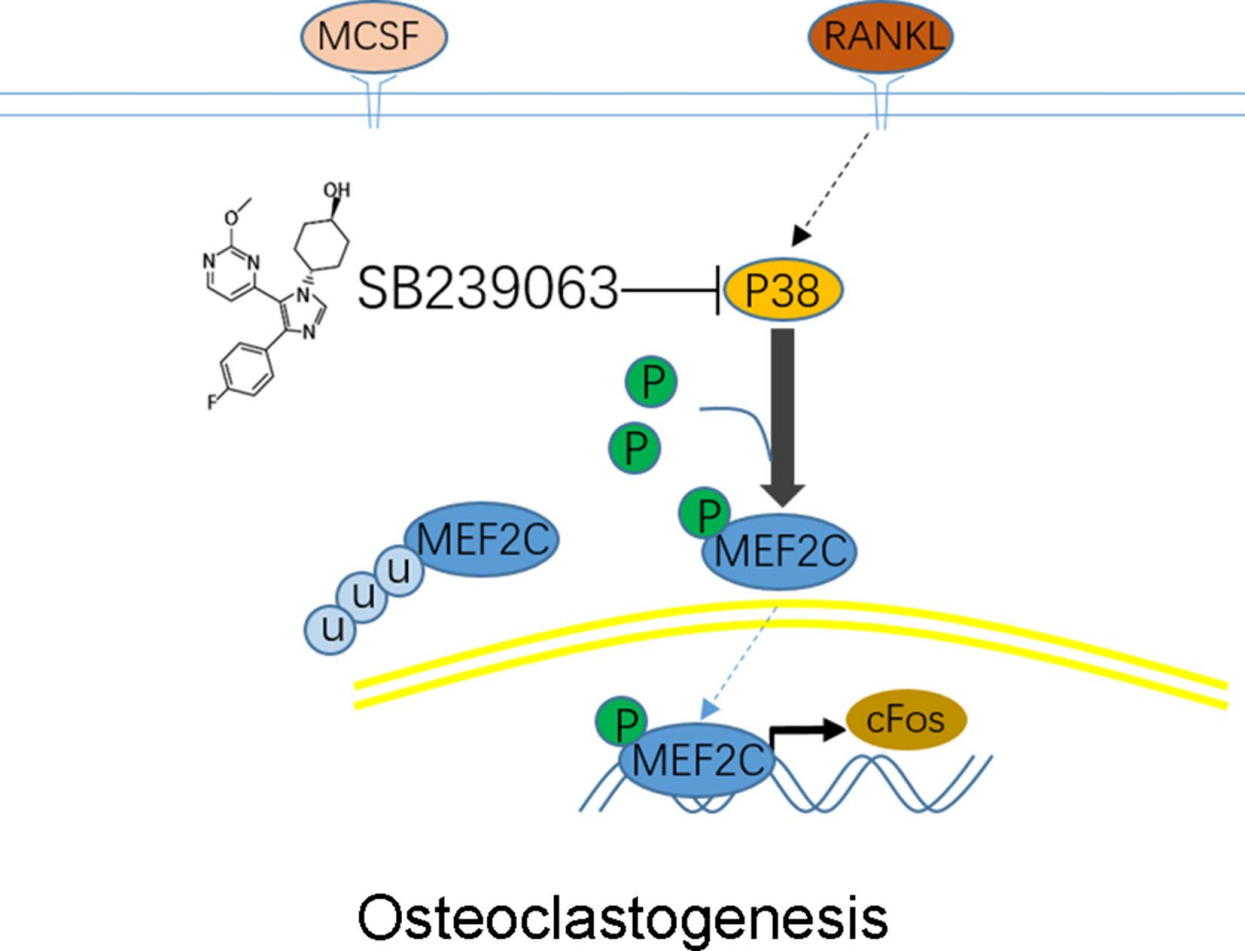
Schematic representation of experiments. Osteoclastogenic signaling cascade controlled by SB239063, inhibitors of p38 mitogen-activated protein kinases (MAPK). Arrows indicate activation of the signaling pathways, while T bars indicate inhibition of the signaling pathways.

We further explored the role of SB239063 treatment in the mouse OVX-induced osteoporosis model. Osteoporosis models were successfully established by assessing uterine and body weight in the groups (**Figures S3A, S3B**). The OVX mice treated with the SB239063 have lower body weight than the vehicle controls (although not less than the shams) (**Figure S3B**). These mice were treated for 8 weeks following OVX operation. Micro-computed tomography (micro-CT) showed extensive bone loss in distal femurs in the groups (**Figure 5A**). BV/TV (bone volume/tissue volume), BS/BV (bone surface/bone volume), Tb.Sp (trabecular separation), Tb.Th (trabecular thickness), and Tb.N (trabecular number) were measured according to three-dimensional (3D) reconstructed images. The value of BV/TV, Tb.Th, and Tb.N significantly decreased, while the value of BS/BV and Tb.Sp increased in distal femurs of mice in vehicle group (**Figures 5B–F**). In contrast, elevated bone mass was detected in SB239063 treatment group compared with that in the vehicle group alone (**Figures 5B–F**). Furthermore, the protective effect of SB239063 on OVX-induced bone loss was confirmed by histological analysis. Trabecular number and thickness in vehicle group were notably reduced, while they were reversed in SB239063 treatment group (**Figure 5G**). In addition, the number of osteoclasts in vehicle group was significantly elevated, while it was reduced in the SB239063 treatment group (**Figures 5H, I**). We also demonstrated that SB239063 did not affect the growth plate (GP) in the proximal tibiae (**Figure 5G**). Finally, as shown in **Figure 5J**, higher expression of MEF2C occurred in lumbar vertebral bodies (L3–L5) of the vehicle group in comparison with that of the sham group. However, in the SB239063-treated group, MEF2C expression could be reversed compared with that in the vehicle group. Finally, schematic representation of osteoclastogenic signaling cascade controlled by SB239063, inhibitors of p38/MAPK, was presented (**Figure 6**). Taken together, administration of SB239063 prevents LPS- and OVX-induced bone loss* in vivo*.

## Discussion

RANKL induced osteoclast formation, and subsequent bone resorption is essential for bone homeostasis. However, osteolytic diseases such as osteoporosis can result from hyperactivity or increased osteoclast formation. Understanding the underlying mechanisms that regulates osteoclast formation is important for future treatment. In the present study, we demonstrated that MEF2C mainly phosphorylated by p38 played a crucial role in osteoclastogenesis by promoting the translational activation of c-Fos. Based on this hypothesis, we showed that administration of SB239063, a novel p38/MAPK-specific inhibitor, could protect bone tissue against LPS-induced bone loss and OVX-induced osteoporosis.

Although more and more compounds that influence the MAPK pathway can affect the osteoclastogenesis through transcription factor c-Fos (Deepak et al., 2015; Chawalitpong et al., 2016; Wei et al., 2018), there are few evidences that show the explicit mechanism through which the p38-specific inhibitor impairs the differentiation of osteoclasts. While p38-mediated signaling pathways are required for inducing osteoclast differentiation (Li et al., 2002), transcriptional activity of c-Fos regulated by p38 still remains unclear. Furthermore, phosphorylation of MEF2C plays an important role in all kinds of tissues (Okamoto et al., 2000; Zhang et al., 2014; Bai et al., 2015; Brown et al., 2018). The p38, not ERK or JNK, can directly phosphorylate MEF2C to initiate nuclear translocation (Han et al., 1997; Okamoto et al., 2000; de Angelis et al., 2005). Therefore, we firstly demonstrated that activation of p38 by RANKL could phosphorylate MEF2C (Ser396) during osteoclast formation. Because ERK (Hu et al., 2017; Wang et al., 2018) and NF-κB (Vaira et al., 2008; Li et al., 2011; Xie et al., 2018) signaling pathways play vital roles in osteoclastogenesis, SB239063 uncommonly affecting the ERK and NF-κB pathways can inhibit osteoclastogenesis by restraining nuclear translocation of MEF2C. P38 and MEF2C mainly work at the early stage in RANKL-induced osteoclasts formation and function, indicating a very close relationship of p38-MEF2C axis.

The activating protein-1 (AP-1) consisted of c-Fos and c-Jun. The c-Fos is regarded as an essential regulator of osteoclastogenesis (Takayanagi et al., 2002b). c-Fos is vital for the expression of osteoclast-associated genes (TRAP, CTSK, and DC-STAMP) (Takayanagi et al., 2002b; Monje et al., 2005). In this study, we discovered that c-Fos expression at the mRNA and protein levels was distinctly downregulated following siMEF2C or SB239063 treatment. In addition to the role of p38/MEF2C in c-Fos expression, IL-7/IL-7R promotes RANKL-mediated osteoclast formation through c-Fos/c-Jun pathway (Zhao et al., 2018), while IL-6 affects the transforming rate from zygotes to blastocysts by impacting IL-6st, c-Fos, and c-Jun expression mainly through JAK-STAT signaling pathway (Yu et al., 2018). The investigation about IL-6 or JAK-STAT signaling pathway was not proceeded due to a limitation. Although c-Fos expression, regulated by p38, and c-Jun expression, regulated by JNK, could be regulated by different pathways (Maedler et al., 2008; Li et al., 2016; Langfermann et al., 2017), our data showed that SB239063 did not affect the JNK, ERK, or NF-κB signaling pathway. The phosphorylation of JNK, ERK, p65, or IκBα was detected, indicating that the c-Fos expression might be specifically regulated by p38. Furthermore, the translocated MEF2C can bind to the promoter region of c-Fos, inducing osteoclast formation. Additionally, SB239063 treatment suppressed the expression of c-Fos, TRAP, CTSK, DC-STAMP, and NFATc1, while the inhibition of osteoclastogenesis by SB239063 could be partially suppressed by overexpression of MEF2C, indirectly indicating its enhancement of c-Fos expression.

In addition, MEF2C protein level was regulated by p38 activation or inhibition in a MUC4/ErbB2/p38/MEF2C-dependent mechanism. (Zhang et al., 2014) In the present study, the results showed that SB239063 treatment downregulated, whereas RANKL treatment upregulated, MEF2C protein level during osteoclastogenesis. Although SB239063 had little effect on the gene expression of MEF2C, MEF2C definitely increased for the promotion of ubiquitination degradation after SB239063 treatment. Maybe, this phosphorylation site (Ser396) stabilizing MEF2C is one of the vital ubiquitination sites. This finding may explain why the gene expression of MEF2 transcription factors and MEF2-dependent transcription activity is inconsistent (Naya et al., 1999). We encountered technical limitations so that post-transcriptional mechanisms of the expression of MEF2C proteins was not conducted. Interestingly, MG132, a proteasome inhibitor, could reverse the suppression of SB239063 treatment on osteoclast formation, further indicating that MEF2C stability is also dependent on its ubiquitination degradation (data not shown). Perhaps, further verification is still required to clarify whether other pathways contributed to reduced MEF2C synthesis or dysfunctional post-transcriptional translation.

Administration of SB239063 exerts an anti-catabolic role in LPS- or OVX-induced bone loss, inhibiting the differentiation and function of osteoclasts *in vivo*. Data are consistent with the inhibition of osteoclast formation and dysfunction of bone resorption treated with SB239063 *in vitro*. In addition, acute lung injury in rat induced by intestinal ischemia reperfusion was alleviated (Xiong et al., 2016), and the neurological status was improved in hepatic encephalopathy (Agusti et al., 2011) through administration of SB239063. Although the anabolic roles of canonical Wnt/β-catenin signaling pathway in the bone were identified (Tu et al., 2015) and MEF2C/β-catenin complex was detected in hepatocellular carcinoma and myoblasts (De Angelis et al., 1998; Bai et al., 2015), siMEF2C or SB239063 was shown to have little effect on anti-/pro-anabolism of osteogenesis *in vitro* (data not shown). MEF2C was increased in OVX-induced osteoporotic intervertebral bodies, whereas this effect could be reversed by SB239063 treatment, in accordance with the reduction of MEF2C protein following SB239063 during RANKL-induced osteoclastogenesis *in vitro*. Finally, inhibition of osteoclastogenesis by SB239063 can also be reversed by overexpression of c-Fos. Nevertheless, our study has certain weaknesses. For instance, further studies can be conducted to explore the function of other MEF2 family including MEF2A, MEF2B, and MEF2D in bone metabolism, so that our mechanistic results can be further verified. SB239063 may be an inhibitor of a ubiquitous pathway that is involved in many processes throughout the body. Therefore, systemic treatment may be expected to produce side effects. Although the underlying mechanisms of the p38/MEF2C/c-Fos axis on bone metabolism were not fully elucidated, these findings are vital to illuminate molecular mechanisms of osteoclastogenesis and osteogenesis.

In conclusion, the p38/MEF2C/c-Fos axis plays an important role in osteoclast differentiation and function. SB239063 can prevent LPS- and OVX-induced bone loss.

## Data Availability

The raw data supporting the conclusions of this manuscript will be made available by the authors, without undue reservation, to any qualified researcher.

## Ethics Statement

All animal experiments were performed in accordance with the guidelines of and were approved by the Institutional Animal Care and Use Committee of KIRAMS.

## Author Contributions

All authors were involved in conception and design. Study design: BH, JC, AQ, SF, and FZ. Study conduct: BH, JW, ZX, and XYZ. Data collection and analysis: JL, XDZ, ZJ, and JW. Data interpretation: AQ, JC, and FZ. Drafting manuscript: BH, HW, and JW. Revising manuscript: BH, AQ, and FZ. All authors take responsibility for the integrity of the data analysis.

## Conflict of Interest Statement

The authors declare that the research was conducted in the absence of any commercial or financial relationships that could be construed as a potential conflict of interest.

## Abbreviations

RANKL, receptor activator of nuclear factor- κB ligand; MAPK, mitogen-activated protein kinase; AP-1, activating protein-1; MEF2C, myocyte-specific enhancer factor 2C; CCK-8, cell counting kit-8; ChIP, chromatin immunoprecipitation; LPS, lipopolysaccharide; OVX, ovariectomy; CTSK, cathepsin K.

## References

[B1] Agusti A. Cauli O. Rodrigo R. Llansola M. Hernandez-Rabaza V. Felipo V. (2011). p38 MAP kinase is a therapeutic target for hepatic encephalopathy in rats with portacaval shunts. Gut 60, 1572-1579. 10.1136/gut.2010.236083 21636647

[B2] Arai F. Miyamoto T. Ohneda O. Inada T. Sudo T. Brasel K. (1999). Commitment and differentiation of osteoclast precursor cells by the sequential expression of c-Fms and receptor activator of nuclear factor kappaB (RANK) receptors. J. Exp. Med. 190, 1741–1754. 10.1084/jem.190.12.1741 10601350 PMC2195707

[B3] Ash P. Loutit J. Townsend K. (1980). Osteoclasts derived from haematopoietic stem cells. Nature 283, 669–670. 10.1038/283669a0 7354855

[B4] Bai X. L. Zhang Q. Ye L. Y. Liang F. Sun X. Chen Y. (2015). Myocyte enhancer factor 2C regulation of hepatocellular carcinoma *via* vascular endothelial growth factor and Wnt/beta-catenin signaling. Oncogene 34, 4089–4097. 10.1038/onc.2014.337 25328135

[B5] Borghi S. Molinari S. Razzini G. Parise F. Battini R. Ferrari S. (2001). The nuclear localization domain of the MEF2 family of transcription factors shows member-specific features and mediates the nuclear import of histone deacetylase 4. J. Cell Sci. 114, 4477–4483. 11792813 10.1242/jcs.114.24.4477

[B6] Brown F. C. Still E. Koche R. P. Yim C. Y. Takao S. Cifani P. (2018). MEF2C phosphorylation is required for chemotherapy resistance in acute myeloid leukemia. Cancer Discov. 8, 478–497. 10.1158/2159-8290.CD-17-1271 29431698 PMC5882571

[B7] Cante-Barrett K. Pieters R. Meijerink J. P. (2014). Myocyte enhancer factor 2C in hematopoiesis and leukemia. Oncogene 33, 403–410. 10.1038/onc.2013.56 23435431

[B8] Chawalitpong S. Sornkaew N. Suksamrarn A. Palaga T. (2016). Diarylheptanoid from Curcuma comosa Roxb. suppresses RANKL-induced osteoclast differentiation by decreasing NFATc1 and c-Fos expression *via* MAPK pathway. Eur. J. Pharmacol. 788, 351–359. 10.1016/j.ejphar.2016.08.012 27523282

[B9] Darnay B. Ni J. Moore P. Aggarwal B. (1999). Activation of NF-kappaB by RANK requires tumor necrosis factor receptor-associated factor (TRAF) 6 and NF-kappaB-inducing kinase. Identification of a novel TRAF6 interaction motif. J. Biol. Chem. 274, 7724–7731. 10.1074/jbc.274.12.7724 10075662

[B10] De Angelis L. Borghi S. Melchionna R. Berghella L. Baccarani-Contri M. Parise F. (1998). Inhibition of myogenesis by transforming growth factor beta is density-dependent and related to the translocation of transcription factor MEF2 to the cytoplasm. Proc. Natl. Acad. Sci. U. S. A. 95, 12358–12363. 10.1073/pnas.95.21.12358 9770491 PMC22836

[B11] De Angelis L. Zhao J. Andreucci J. J. Olson E. N. Cossu G. McDermott J. C. (2005). Regulation of vertebrate myotome development by the p38 MAP kinase-MEF2 signaling pathway. Dev. Biol. 283, 171–179. 10.1016/j.ydbio.2005.04.009 15890335

[B12] Deepak V. Kruger M. C. Joubert A. Coetzee M. (2015). Piperine alleviates osteoclast formation through the p38/c-Fos/NFATc1 signaling axis. BioFactors 41, 403–413. 10.1002/biof.1241 26627060

[B13] Feng H. Cheng T. Steer J. H. Joyce D. A. Pavlos N. J. Leong C. (2009). Myocyte enhancer factor 2 and microphthalmia-associated transcription factor cooperate with NFATc1 to transactivate the V-ATPase d2 promoter during RANKL-induced osteoclastogenesis. J. Biol. Chem. 284, 14667–14676. 10.1074/jbc.M901670200 19321441 PMC2682914

[B14] Han J. Jiang Y. Li Z. Kravchenko V. Ulevitch R. (1997). Activation of the transcription factor MEF2C by the MAP kinase p38 in inflammation. Nature 386, 296–299. 10.1038/386296a0 9069290

[B15] Hill C. Treisman R. (1995). Transcriptional regulation by extracellular signals: mechanisms and specificity. Cell 80, 199–211. 10.1016/0092-8674(95)90403-4 7834740

[B16] Hu X. Ping Z. Gan M. Tao Y. Wang L. Shi J. (2017). Theaflavin-3,3’-digallate represses osteoclastogenesis and prevents wear debris-induced osteolysis *via* suppression of ERK pathway. Acta. Biomater. 48, 479–488. 10.1016/j.actbio.2016.11.022 27838465

[B17] Karin M. Liu Z. Zandi E. (1997). AP-1 function and regulation. Curr. Opin. Cell Biol. 9, 240–246. 10.1016/S0955-0674(97)80068-3 9069263

[B18] Kramer I. Baertschi S. Halleux C. Keller H. Kneissel M. (2012). Mef2c deletion in osteocytes results in increased bone mass. J. Bone Miner. Res. 27, 360–373. 10.1002/jbmr.1492 22161640

[B19] Langfermann D. S. Rossler O. G. Thiel G. (2017). Stimulation of B-Raf increases c-Jun and c-Fos expression and upregulates AP-1-regulated gene transcription in insulinoma cells. Mol. Cell. Endocrinol. 472, 126–139. 10.1016/j.mce.2017.12.003 29225069

[B20] Li C. Yang Z. Li Z. Ma Y. Zhang L. Zheng C. (2011). Maslinic acid suppresses osteoclastogenesis and prevents ovariectomy-induced bone loss by regulating RANKL-mediated NF-kappaB and MAPK signaling pathways. J. Bone Miner. Res. 26, 644–656. 10.1002/jbmr.242 20814972

[B21] Li J. K. Nie L. Zhao Y. P. Zhang Y. Q. Wang X. Wang S. S. (2016). IL-17 mediates inflammatory reactions *via* p38/c-Fos and JNK/c-Jun activation in an AP-1-dependent manner in human nucleus pulposus cells. J. Transl. Med. 14, 77. 10.1186/s12967-016-0833-9 26988982 PMC4794827

[B22] Li X. Udagawa N. Itoh K. Suda K. Murase Y. Nishihara T. (2002). p38 MAPK-mediated signals are required for inducing osteoclast differentiation but not for osteoclast function. Endocrinology 143, 3105–3113 10.1210/endo.143.8.8954 12130576

[B23] Maedler K. Schulthess F. T. Bielman C. Berney T. Bonny C. Prentki M. (2008). Glucose and leptin induce apoptosis in human beta-cells and impair glucose-stimulated insulin secretion through activation of c-Jun N-terminal kinases. FASEB J. 22, 1905–1913. 10.1096/fj.07-101824 18263705

[B24] Monje P. Hernandez-Losa J. Lyons R. J. Castellone M. D. Gutkind J. S. (2005). Regulation of the transcriptional activity of c-Fos by ERK. A novel role for the prolyl isomerase PIN1. J. Biol. Chem. 280, 35081–35084. 10.1074/jbc.C500353200 16123044

[B25] Nakatani T. Partridge N. C. (2017). MEF2C interacts with c-FOS in PTH-stimulated Mmp13 gene expression in osteoblastic cells. Endocrinology 158, 3778–3791. 10.1210/en.2017-00159 28973134 PMC5695834

[B26] Naya F. Wu C. Richardson J. Overbeek P. Olson E. (1999). Transcriptional activity of MEF2 during mouse embryogenesis monitored with a MEF2-dependent transgene. Development 123, 2045–2052. 10.1242/dev.126.10.2045 10207130

[B27] Okamoto S. Krainc D. Sherman K. Lipton S. A. (2000). Antiapoptotic role of the p38 mitogen-activated protein kinase-myocyte enhancer factor 2 transcription factor pathway during neuronal differentiation. Proc. Natl. Acad. Sci. U. S. A. 97, 7561–7566. 10.1073/pnas.130502697 10852968 PMC16585

[B28] Pearson G. Robinson F. Beers Gibson T. Xu B. E. Karandikar M. Berman K. (2001). Mitogen-activated protein (MAP) kinase pathways: regulation and physiological functions. Endocr. Rev. 22, 153–183. 10.1210/er.22.2.153 11294822

[B29] Scheven B. Visser J. Nijweide P. (1986). In vitro osteoclast generation from different bone marrow fractions, including a highly enriched haematopoietic stem cell population. Nature 321, 79–81. 10.1038/321079a0 3754620

[B30] Takayanagi H. Kim S. Koga T. Nishina H. Isshiki M. Yoshida H. (2002a). Induction and activation of the transcription factor NFATc1 (NFAT2) integrate RANKL signaling in terminal differentiation of osteoclasts. Dev. Cell 3, 889–901. 10.1016/S1534-5807(02)00369-6 12479813

[B31] Takayanagi H. Kim S. Matsuo K. Suzuki H. Suzuki T. Sato K. (2002b). RANKL maintains bone homeostasis through c-Fos-dependent induction of interferon-beta. Nature 416, 744–749. 10.1038/416744a 11961557

[B32] Treisman R. (1996). Regulation of transcription by MAP kinase cascades. Curr. Opin. Cell Biol. 8, 205–215. 10.1016/S0955-0674(96)80067-6 8791420

[B33] Tu X. Delgado-Calle J. Condon K. W. Maycas M. Zhang H. Carlesso N. (2015). Osteocytes mediate the anabolic actions of canonical Wnt/β-catenin signaling in bone. Proc. Natl. Acad. Sci. U. S. A. 112, E478–E486. 10.1073/pnas.1409857112 25605937 PMC4321271

[B34] Vaira S. Alhawagri M. Anwisye I. Kitaura H. Faccio R. Novack D. V. (2008). RelA/p65 promotes osteoclast differentiation by blocking a RANKL-induced apoptotic JNK pathway in mice. J. Clin. Invest. 118, 2088–2097. 10.1172/JCI33392 18464930 PMC2373419

[B35] Wang L. Iorio C. Yan K. Yang H. Takeshita S. Kang S. (2018). A ERK/RSK-mediated negative feedback loop regulates M-CSF–evoked PI3K/AKT activation in macrophages. FASEB J. 32, 875–887. 10.1096/fj.201700672RR 29046360 PMC5888401

[B36] Wei C. M. Liu Q. Song F. M. Lin X. X. Su Y. J. Xu J. (2018). Artesunate Inhibits RANKL-induced osteoclastogenesis and bone resorption in vitro and prevents lps-induced bone loss *in vivo*. J. Cell. Physiol. 233, 476–485. 10.1002/jcp.25907 28294321

[B37] Xie Z. Yu H. Sun X. Tang P. Jie Z. Chen S. (2018). A novel diterpenoid suppresses osteoclastogenesis and promotes osteogenesis by inhibiting Ifrd1-mediated and ikappabalpha-mediated p65 nuclear translocation. J. Bone Miner. Res. 33, 667-678. 10.1002/jbmr.3334 29091322

[B38] Xiong L. L. Tan Y. Ma H. Y. Dai P. Qin Y. X. Yang R. A. (2016). Administration of SB239063, a potent p38 MAPK inhibitor, alleviates acute lung injury induced by intestinal ischemia reperfusion in rats associated with AQP4 downregulation. Int. Immunopharmacol. 38, 54–60. 10.1016/j.intimp.2016.03.036 27236300

[B39] Yu C. Zhang X. Wang L. Liu Y. Li N. Li M. (2018). Interleukin-6 regulates expression of Fos and Jun genes to affect the development of mouse preimplantation embryos. J. Obstet. Gynaecol. Res. 44, 253–262. 10.1111/jog.13498 29171142 PMC5836979

[B40] Zhang J. J. Zhu Y. Xie K. L. Peng Y. P. Tao J. Q. Tang J. (2014). Yin Yang-1 suppresses invasion and metastasis of pancreatic ductal adenocarcinoma by downregulating MMP10 in a MUC4/ErbB2/p38/MEF2C-dependent mechanism. Mol. Cancer 13, 130. 10.1186/1476-4598-13-130 24884523 PMC4047260

[B41] Zhao J. J. Wu Z. F. Yu Y. H. Wang L. Cheng L. (2018). Effects of interleukin-7/interleukin-7 receptor on RANKL-mediated osteoclast differentiation and ovariectomy-induced bone loss by regulating c-Fos/c-Jun pathway. J. Cell. Physiol. 233, 7182–7194. 10.1002/jcp.26548 29663382 PMC13482568

