# Supplementary material for: Administration of SB239063 Ameliorates Ovariectomy-Induced Bone Loss via Suppressing Osteoclastogenesis in Mice
Source: Front Pharmacol. 2019 Aug 15;10:900. doi: 10.3389/fphar.2019.00900 (PMC6704231; doi:10.3389/fphar.2019.00900)
Supplement: Supplementary file 1 [file DataSheet_1.pdf]

Supplemental Figure Legends

Suppl Figure 1

(A)The molecular structure of SB239063. (B-C) Effect of SB239063 on BMMs viability at (B)48h and (C)96h. (D) The viral transduction of pLenti-EF1a-MEF2C or the empty vector in BMMs. (E) The viral transduction of pLenti-EF1a-c-Fos or the empty vector in BMMs. (F)The number of TRAP-positive BMMs following the viral transduction of pLenti-EF1a-c-Fos or the empty vector and the treatment with SB239063. All experiments were performed at least three times. Scale bar, 100 μm. *P<0.05, **P<0.01, ***P<0.005, compared with the untreated cells.

Suppl Figure 2

(A-B) Cell viability of SB2339063-treated MC3T3-E1 cells at (A)48h or (B)96 h.

Suppl Figure 3

(A)The uteruses and (B)body weight of three groups were presented after OVX operation treated for 8 weeks. *P<0.05, **P<0.01, ***P<0.005.

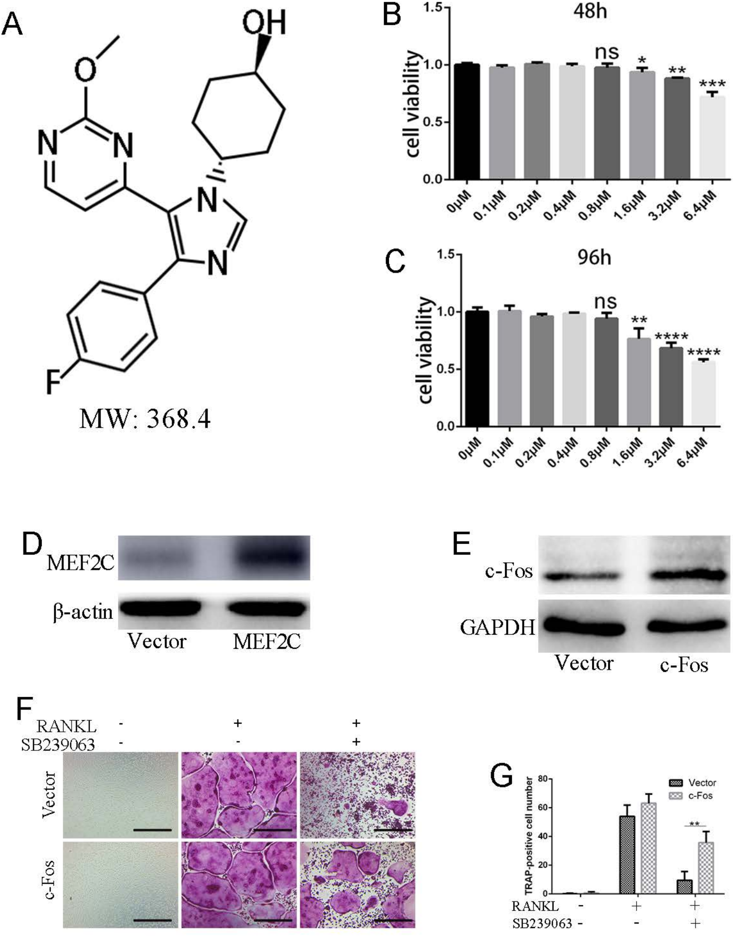
A
OH
O
N
N
N
N
F
MW: 368.4
B
48h
cell viability
1.5
1.0
0.5
0.0
ns
*
**
***
0μM
0.1μM
0.2μM
0.4μM
0.8μM
1.6μM
3.2μM
6.4μM
C
96h
cell viability
1.5
1.0
0.5
0.0
ns
**
****
****
0μM
0.1μM
0.2μM
0.4μM
0.8μM
1.6μM
3.2μM
6.4μM
D
MEF2C
β-actin
Vector
MEF2C
E
c-Fos
GAPDH
Vector
c-Fos
F
RANKL - + +
SB239063 - - +
Vector
c-Fos
G
TRAP-positive cell number
80
60
40
20
0
Vector
c-Fos
**
RANKL - + +
SB239063 - - +

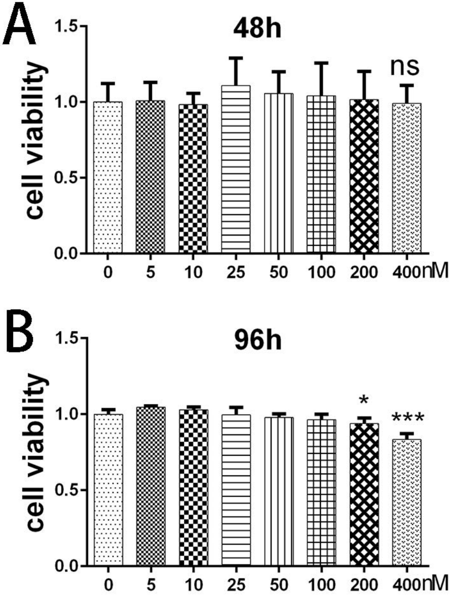
A
48h
cell viability
1.5
1.0
0.5
0.0
ns
0
5
10
25
50
100
200
400nM
B
96h
cell viability
1.5
1.0
0.5
0.0
*
***
0
5
10
25
50
100
200
400nM

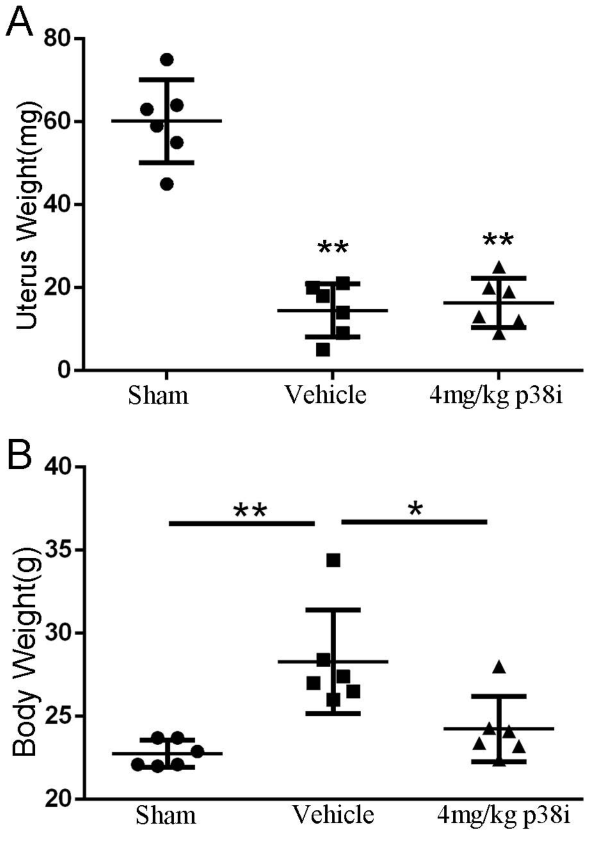
A
80
60
40
20
0
Uterus Weight(mg)
**
**
Sham
Vehicle
4mg/kg p38i
B
40
35
30
25
20
Body Weight(g)
**
*
Sham
Vehicle
4mg/kg p38i

Supplementary Table 1 Sequences of primers

Gene	Primer sequences (5'-3')	
c-Fos	Forward	GGGAATGGTGAAGACCGTGT
	Reverse	CCGTTCCCTTCGGATTCTCC
NFATc1	Forward	CCCGTCACATTCTGGTCCAT
	Reverse	CAAGTAACCGTGTAGCTGCACAA
CTSK	Forward	GCTCACAGTAGCCACGCTT
	Reverse	AACGCCGAGAGATTTCATCCA
TRAP	Forward	AAGAGATCGCCAGAACCGTG
	Reverse	TTCCAGCCAGCACATACCAG
MEF2C	Forward	ATCACGCATCTCACCGCTTG
	Reverse	AGGAAGTTGTTCCCGTCAGC
GAPDH	Forward	GGAGAGTGTTTCCTCGTCCC
	Reverse	ATGAAGGGGTCGTTGATGGC
β-actin	Forward	GGCTGTATTCCCCTCCATCG
	Reverse	CCAGTTGGTAACAATGCCATGT

Supplementary Table 2 Target Gene Information

Name	Sequence Information
siMEF2C	#1:GCATTGAACAAGAAAGAAA<br>#2:GGAAATTTGGATTGATGAA<br>#3:CAAGAATATACAAGCCAAA
MEF2C	Mouse NM_025282.3
c-Fos	Mouse NM_010234.3
